# Pharmacological effects of *Radix Angelica Sinensis *(*Danggui*) on cerebral infarction

**DOI:** 10.1186/1749-8546-6-32

**Published:** 2011-08-25

**Authors:** Yi-Chian Wu, Ching-Liang Hsieh

**Affiliations:** 1 Department of Chinese Medicine, China Medical University Hospital, Taichung 40402, Taiwan; 2 Graduate Institute of Acupuncture Science, China Medical University, Taichung 40402, Taiwan; 3 Acupuncture Research Center, China Medical University, Taichung 40402, Taiwan

## Abstract

*Radix Angelica Sinensis*, the dried root of *Angelica sinensis *(*Danggui*), is a herb used in Chinese medicine to enrich blood, promote blood circulation and modulate the immune system. It is also used to treat chronic constipation of the elderly and debilitated as well as menstrual disorders. Research has demonstrated that *Danggui *and its active ingredients, as anti-arthrosclerotic, anti-hypertensive, antioxidant anti-inflammatory agents which would limit platelet aggregation, are effective in reducing the size of cerebral infarction and improving neurological deficit scores.

## Background

*Danggui*, the dried root of *Angelica Sinensis *(*Radix Angelica Sinensis*), is a commonly used Chinese medicinal herb to enrich blood, promote blood circulation and treat blood deficiency pattern and menstrual disorders such as dysmenorrhea and irregular menstrual cycle [1]. Wilasrusmee *et al. *[2] reported that *Danggui *(105 μg/ml) plays an immunostimulatory role in mitogen-stimulated murine lymphocytes *in vitro*. Angelan, a purified polysaccharide component of *Angelica nakai *thought to improve immune function, increases the expression of cytokines in splenocytes as Angelan enhances and the production of interleukin-6 (IL-6) and interferon-γ(IFN-γ) of activated macrophages, helper T cells and natural killer cells [3]. The chemical constituents of the *Danggui *extract are classified into essential oil and water soluble parts including lipid compounds, phenolic compounds, carbohydrates, organic acids and other constituents [4]. The most active ingredients are polysaccharides, Z-Ligustilide (3-butylidene-4,5-dihydrophthalide) and ferulic acid (4-hydroxy-3-methoxycinnamicacid) [1].

This article aims to provide an overview of the pharmacological effects of *Danggui *in reducing the size of cerebral infarction and improving neurological deficit scores.

### Search strategy

We searched Medline, PubMed, Cochrane Library and the China National Knowledge Infrastructure (Chinese language database) between 1990 and 2010, using '*Angelica sinensis*', '*Danggui*', '*Angelica *polysaccharides', 'Z-Ligustilide', 'Ferulic acid' and 'Ischemic stroke' as keywords.

### Vasodilation and improving microcirculation

Nitric oxide (NO) is synthesized with nitric oxide synthase (NOS) which includes three different isoforms, namely endothelial NOS (eNOS), neuronal NOS (nNOS) and inducible NOS (iNOS) [5]. While nNOS and eNOS are induced under different conditions, their activation relies on intracellular Ca^2+ ^for binding calmodulin [5,6]. Due to its vasodilative effects, eNOS is considered neuro-protective [6]. Hypertension and a lack of endothelium-derived relaxing factor activity are found in eNOS knockout mice [7]. Moreover, the cerebral infarction size is larger in a model of eNOS mutant mice with middle cerebral artery occlusion (MCAo) [8]. Therefore, eNOS has a vasodilation effect and is neuro-protective by increasing the blood flow [9]. It is possible that *Danggui *increases NO formation and relaxes the endothelium [10], thereby limiting infarction size. In rabbits on a high-lipid diet, treatment with ferulic acid, an active component of *Danggui*, increases the generation of NO, thereby inhibiting platelet aggregation on endothelium and proliferation of smooth muscles and preventing leucocytes adhering to the endothelium [11].

Z-Ligustilide (3-butylidene-4,5-dihydrophthalide), a component of *Danggui*, inhibits (4-8 μg/ml) the spontaneous contraction of isolated rat uterus in a dose-dependent manner [12]. Moreover, Z-Ligustilide may also inhibit prostaglandin F-2α, oxytocin, acetylcholine chloride and potassium depolarization-induced uterine contraction, suggesting that Ligustilide modulates the function of uterine tissue and has a non-specific anti-spasmodic effect [12]. Z-Ligustilide enhances the recovery of conjunctival capillary and venue diameter after dextran T500 administration to rabbits and increases the number of opened capillaries as well as blood flow, suggesting that Z-Ligustilide improves microcirculation [13].

Ferulic acid is the main organic acid component of *Danggui*. Ferulic acid (10^-3 ^mol/L) relaxes the phenylephrine-induced contraction of aorta ring in spontaneous on rat (SHR) whereas the effects of ferulic acid may be partially blocked by pretreatment of the aorta with *N*^G^-nitro-L-arginine methyl ester (L-NAME, 10^-4 ^mol/L) which inhibits the production of NO from L-arginine [14]. Ferulic acid (10^-3 ^mol/L) reduces the production of thromboxane B_2 _in the aorta ring of SHR [14]. Ferulic acid (10^-4 ^mol/L) also significantly reduces the generation of NADPH-dependent production of the superoxide anion [14] and enhances the acetylcholine-induced vasodilation whereas hydroxyhydroquinone (HHQ) inhibits this effect [14]. Taken together, ferulic acid reduces blood pressure in SHR *via *effects on (1) eNOS; (2) the inhibition of thromboxane B_2 _to relax aorta ring; (3) reactive oxygen species (ROS) scavenging activity to increase the availability of NO in endothelial cell of aorta [14].

### Anti-arthrosclerosis effects

Stroke is divided into two major groups according to the cerebral damage, cerebral infarction and cerebral hemorrhage. Eighty percent (80%) of stroke patient suffer from cerebral infarction [15]. Cerebral infarction is mainly caused by thrombosis, embolism or systemic hemodynamic hypertension. Atherosclerosis in large and small arteries is a major contributor to cerebral thrombosis. The etiology of atherosclerosis and stroke is related to inflammation and genetic factors. Ischemic cerebral infarction may be prevented by anti-inflammatory agents or the treatment of vascular diseases, heart diseases and hypertension [16-18].

A principal contributor to cerebral infarction and atherosclerosis is believed to be initiated by an excessive inflammatory-fibro-proliferative response [19]. Atherosclerosis involves growth factors, cytokines and vaso-regulatory factors such as vascular endothelial growth factor (VEGF), fibroblast growth factor (FGH), transforming growth factor-β (TGF-β), interleukin-1 (IL-1) and tumor necrosis factor -α (TNF-α) [19,20]. Cytokines are both pro- and anti-atherogenic; for example, IL-1 and TNF-α mediate the production of monocyte chemoattractant protein-1 (MCP-1) to induce monocyte migration directly into the intima. By contrast, cytokines can induce NO production which regulates the vasomotor tone of artery, thereby influencing the initiation and progression of the atherosclerosis process [20]. A previous study [21] found that nicotine can mediate the development and progression of atherosclerosis *via *the inhibition of TGF-β1 and basic fibroblast growth factor (bFGF). The alteration of TGB-β activity leads to the atherosclerotic change of vessel wall and increased TGB-β signaling plays a protective role of atherosclerosis [22]. A study [23] reported that bFGF enhances smooth muscle migration and proliferation *via *the regulation of interstitial collagenase expression in the early stages of atherosclerosis. Wang *et al. *[24] found that the levels of TGB-β reduced and those of bFGF increased in human umbilical vein endothelial cells damaged by hyperlipidemic serum. Moreover, under electromicroscopy the morphology of endothelial cell was also damaged which was reversed by *Danggui *(20 mg/ml) and its component of sodium ferulate (0.3 mg/ml). These results indicate that both *Danggui *and sodium ferulate have anti-atherogenic effects [24]. Yu *et al. *[25] found that the levels of total cholesterol (TC, 0.95 mmol/L *vs*. 11.79 mmol/L), triglyceride (TG) and high density lipoprotein cholesterol (HDLC) and low density lipoprotein cholesterol (LDLC) increased in rabbits on a high-lipid diet compared to the control group which was on a normal diet. After 25% *Danggui *was administered (i.v.) for four weeks, the levels of TG decreased from 3.52 mmol/L to 1.68 mmol/L. The plaque area of thoracic aorta was also reduced after *Danggui *treatment, from 63.31% to 35.58% [25]. Moreover, *Danggui *reduced the increase of the serum malonyldialdehyde (MDA) levels caused by the high-lipid diet [25]. In a similar study [11], after treatment with sodium ferulate, the plaque area of thoracic aorta was reduced while the TG level was reduced to 1.75 mmol/L in rabbits on a high-lipid diet [11]. Moreover, the sodium ferulate-treated group increased the production of NO from epithelium cells. Both *Danggui *and sodium ferulate inhibit the formation of atherosclerosis because *Danggui *reduces the TG and lipid peroxidation levels or increases NO production, or both.

### Anti-platelet aggregation effects

Anti-platelet aggregation agents such as aspirin, ticlopidine and clopidogrel are widely used to prevent secondary ischemic stroke [16,26]. A clinical trial [26] reported that administration of aspirin within six hours of the onset of ischemic stroke reduces the patients' mortality rate.

*Danggui *dose-dependently inhibited adenosine diphosphate (ADP)-induced platelet aggregation and collagen-induced platelet [27]. *Danggui *(20 g/kg) reduced platelet aggregation by 87.9% in rats with ADP-induced platelet aggregation and 33.0% in rats with collagen-induced platelet aggregation [27]. Administration of sodium ferulate (0.2 g/kg, i.v.) reduced ADP-induced platelet aggregation by 38% in rats while a lower dose (0.1 g/kg) reduced collagen-induced platelet aggregation by 81% [27]. In an arteriovenous shunt rat model, the wet weight of the thrombus was reduced to 19.5 mg (46.4 mg in the control) in rats administered with Z-Ligustilide (10 mg/kg, p.o.) and 13.6 mg in rats administered with more Z-Ligustilide (40 mg/kg, p.o.) [28]. The maximal platelet aggregation was 6.8% and 2.0% in the 10 mg/kg and 40 mg/kg groups respectively while the control was 44.6% [28]. Compared with warfarin (1.0 mg/kg, p.o.), Z-Ligustilide (10 mg/kg and 40 mg/kg) administered orally for three days did not increase activated partial thromboplastin time (APTT) and prothrombin time (PT) in a coagulation time test *ex vivo *[28]. *Danggui *and Z-Ligustilide exert anti-platelet aggregation effects.

### Anti-inflammatory effects

Pro-inflammatory cytokines, such as IL-1β and TNF-α, are increased in the brain tissue of transient middle cerebral artery occlusion (MCAo) rats [29,30]. IL-1 up-regulates the expression of adhesion molecules such as intercellular adhesion molecule-1 (ICAM-1), P-selectin and E-selectin in the endothelium [31,32]. These adhesion molecules facilitate the translocation of activated leukocytes into the ischemic core [31,32]. Moreover, nuclear factor-κB (NF-κB) is also activated in the ischemic core [32]. Both *Sophora japonica *L. and paeoniflorin inhibit IL-1β secretion, thereby reducing cerebral infarction size and neurological deficit [29]. Moreover, paeoniflorin reduces IL-1β, TNF-α, ICAM-1 and leucocytes [30]. Therefore, anti-inflammation, such as inhibition of pro-inflammatory cytokine and ICAM-1, is very important in treating cerebral infarction.

Ferulic acid (*eg *80 and 100 mg/kg, i.v.) reduces the size of cerebral infarction and neurological deficit scores and inhibits ICAM-1 and NF-κB expression in transient MCAo rats [33]. The anti-inflammatory action of ferulic acid is, at least in part, important in its therapeutic effect on cerebral infarct [33]. Moreover, ferulic acid (*eg *100 mg/kg, i.v.) exerts anti-inflammatory action by reducing the generation of 4-hydroxy-2-nonenal (4-HNE), 8-hydroxy-2'-deoxyguanosine (8-OHdG) and apoptosis in the reperfusion period after cerebral ischemia, thus providing neuroprotection [32]. This neuro-protection by ferulic acid is thought to occur *via *enhancing gamma-aminobutyric acid type B1 (GABA_B1_) receptor expression to against p38 mitogen activated protein kinase (MAPK)-mediated NO-induced apoptosis [34]. *Danggui *reduces inflammatory cell infiltration and TNF-α and TGF-ß1 mRNA expression; it also reduces TNF-α and TGF-ß1 positive cells in radiation-induced pneumonitis in mice [35]. *Danggui *polysaccharides reduce TNF-α levels in the colon mucosa during intra-colon enema with 2,4,6-trinitrobenzene sulfonic acid (TNBS) and ethanol in rats [36]. Both *Danggui *and ferulic acid exert anti-inflammatory effects.

### Anti-oxidative effects

Reactive oxygen species (ROS) including the superoxide anion, hydrogen peroxide and hydroxyl radical are generated after cerebral ischemia. The ROS affect mitochondrial function, DNA repair and transcription factors, leading to apoptosis [37,6]. Superoxide dismutase 1 (SOD1), an endogenous antioxidant, blocks the early release of cytochrome c from mitochondria and reduces the development of apoptosis in focal cerebral ischemic mice [38]. Apolipoprotein E, *via *its anti-oxidative effects against cerebral ischemia, is neuro-protective in transient forebrain ischemia induced by bilateral common carotid artery occlusion (BCCAo) in mice [39]. Anti-oxidant nutrients such as vitamin E and *Ginkgo biloba *extract reduce cerebral damage in rodent models of ischemia and reperfusion [40]. GABA_B _receptor agonist baclofen may be neuro-protective *via *the inhibition of N-methyl-D-asparate (NMDA) receptor-mediated NO production in brain ischemic injury [41]. Ferulic acid (100 mg/kg, i.v.) enhanced the expression of GABA_B1 _in the reperfusion period (three and 24 hours after ischemia) in rats [34]. Z-ligustilide reduced the size of cerebral infarction from 22.1% to 11.8% (5 mg/kg, i.p.) and 2.60% (20 mg/kg, i.p.) [42]. Z-ligustilide reduced the MDA levels and increased glutathione peroxidase (GSH-Px) and SOD activities in the ischemia-reperfusion brain tissues induced by BCCAo in mice [42]. *Danggui *or its components or both demonstrate anti-oxidant activity in ischemia-reperfusion injury models.

### Effect of *Danggui *on cerebral infarction

*Danggui *(5 g/kg, i.p.) increased blood circulation and neuronal metabolism in an MCAo rat model [43]. *Danggui *reduced the size of cerebral infarction, neurological deficit scores and increased blood flow and SOD activity in the MCAo rat model [44]. Z-ligustilide reduced cerebral infarction size to 10.90% and 3.19% in rats orally dosed with 20 m/kg or 80 mg/kg respectively (21.08% in the control group) in a MCAo model [45]. Moreover, Z-ligustilide (10 mg/kg or 40 mg/kg, p.o.) increased choline acetyltransferase activity and inhibited acetylcholinesterase to improve cognitive function in rats with hypoperfusion [46]. Our previous study [33] found that ferulic acid (80 mg/kg or 100 mg/kg, i.v.) reduced cerebral infarction size and neurological deficit scores in rats. Liu *et al. *[47] reported that *Danggui *(25%, i.v.) administered to 1040 patients with acute cerebral infarction improved neuro-function scores and the Barthel index score more than *Salvia miltiorrhiza *(78.7% *vs*. 59.3%). *Danggui *reduces the size of cerebral infraction and improves neurological deficit scores.

## Conclusion

The effects of *Danggui *on cerebral infarction are through multiple pathways, including anti-arthrosclerosis, improving microcirculation, anti-platelet aggregation, anti-inflammatory and anti-oxidative effects (Table 1). *Danggui *may be useful in treating the cerebral infarction type of stroke.

**Table 1:** Possible pharmacological actions of *Radix Angelica Sinensis *on cerebral infarction

Pharmacological actions	Related components	Possible mechanisms
**Anti-arthrosclerosis effects**	*Danggui *and sodium ferulate	reverse the reduction of TGB-β/reverse the increase of bFGF [24]
	
	*Danggui*	reduce the increase of serum malonyldialdehyde (MDA) levels [25]
	
	sodium ferulated	decrease the levels of triglyceride [11]

**Vasodilatation and improving microcirculation effects**	*Danggui*	increase the formation of NO and mediate the inhibition of calcium influx [10]
	
	sodium ferulate	increase the generation of NO [11]
	
	Ligustilide	inhibit prostaglandin F-2α, oxytocin, acetylcholine chloride, and potassium depolarization-induced muscle contraction [12]
	
	Ligustilide	increase the number of opened capillary and the speed of blood flow [13]
	
	Ferulic acid	enhance acetylcholine-induced vasodilatation and reduce the production of thromboxane B_2 _[14]

**Anti-platelet aggregation effects**	*Danggui *and sodium ferulate	inhibit ADP-induced and collagen-induced platelet aggregation [27]
	
	Z-Ligustilide	inhibit ADP-induced platelet aggregation [28]

**Anti-inflammatory effects**	Ferulic acid	inhibit ICAM-1 and NF-κB expression [33]
	
	Ferulic acid	enhance gamma-aminobutyric acid type B1 (GABA_B1_) receptor expression [34]
	
	*Danggui*	reduce TNF-α and TGF-ß1 mRNA expression [35]
	
	*Danggui *polysaccharides	reduce TNF-α levels [36]

**Anti-oxidative effects**	Ferulic acid	reduce the generation of NADPH-dependent production of superoxide anion [14]
	
	Ferulic acid	enhances the expression of GABA_B1 _receptor expression [34]
	
	Z-ligustilide	reduce MDA levels and increase GSH-PX and SOD activities [42]

## Abbreviations

IL: interleukin; IFN-γ: interferon-γ; AP: polysaccharide component; ALT: serum alanine transferase; MDA: malondialdehyde; NOS: nitric oxide synthase; CCL_4_: carbon tetrachloride; ASP: water-soluble polysaccharide; NO: nitric oxide; eNOS: endothelial nitric oxide synthase; nNOS: neuronal nitric oxide synthase;iNOS: inducible nitric oxide synthase; MCAo: middle cerebral artery occlusion; L-NAME: N^G^-nitro-L-arginine methyl ester; SHR: spontaneous hypertensive rat; NADPH:nicotinamide adenine dinucleotide phosphate; HHQ: hydroxyhydroquinone; ROS: reactive oxygen species; VEGF: vascular endothelial growth factor; FGH: fibroblast growth factor; TGF-β: transforming growth factor-β; TNF-α: tumor necrosis factor -α; MCP-1: monocyte chemoattractant protein-1; bFGF: basic fibroblast growth factor; TC: total cholesterol; TG: triglyceride; HDLC: high density lipoprotein cholesterol; LDLC: low density lipoprotein cholesterol; ADP: adenosine diphosphate; APTT: activated partial thromboplastin time; PT: prothrombin time; ICAM-1: intracellular adhesion molecule-1; NF-κB: nuclear factor-κB; 4-HNE: 4-hydroxy-2-nonenal; 8-OHdG: 8-hydroxy-2'-deoxyguanosine; GABA_B1_: gamma-aminobutyric acid type B1; MAPK: mitogen activated protein kinase; mRNA: messenger ribonucleic acid; SOD: superoxide dismutase; NMDA: N-methyl-D-asparate; GSH-Px: glutathione peroxidase; BCCAo: bilateral carotid artery occlusion

## Competing interests

The authors declare that they have no competing interests.

## Authors' contributions

YCW searched the literature, organized the information and wrote the manuscript. CLH analyzed the information and revised the manuscript. Both authors read and approved the final version of the manuscript.
